# Respiratory Viruses and Bacteria among Pilgrims during the 2013 Hajj

**DOI:** 10.3201/eid2011.140600

**Published:** 2014-11

**Authors:** Samir Benkouiten, Rémi Charrel, Khadidja Belhouchat, Tassadit Drali, Antoine Nougairede, Nicolas Salez, Ziad A. Memish, Malak al Masri, Pierre-Edouard Fournier, Didier Raoult, Philippe Brouqui, Philippe Parola, Philippe Gautret

**Affiliations:** Aix Marseille Université, Marseille, France (S. Benkouiten, R. Charrel, K. Belhouchat, T. Drali, A. Nougairede, N. Salez, P.-E. Fournier, D. Raoult, P. Brouqui, P. Parola, P. Gautret);; Institut Hospitalo-Universitaire Méditerranée, Marseille (S. Benkouiten, R. Charrel, A. Nougairede, N. Salez, P.-E. Fournier, D. Raoult, P. Brouqui, P. Parola, P. Gautret);; Saudi Ministry of Health, Riyadh, Saudi Arabia (Z.A. Memish, M. al Masri);; Alfaisal University College of Medicine, Riyadh (Z.A. Memish)

**Keywords:** bacteria, viruses, cohort study, Hajj, respiratory tract infections, pilgrims, Saudi Arabia

## Abstract

Pilgrims returning from the Hajj might contribute to international spreading of respiratory pathogens. Nasal and throat swab specimens were obtained from 129 pilgrims in 2013 before they departed from France and before they left Saudi Arabia, and tested by PCR for respiratory viruses and bacteria. Overall, 21.5% and 38.8% of pre-Hajj and post-Hajj specimens, respectively, were positive for ≥1 virus (p = 0.003). One third (29.8%) of the participants acquired ≥1 virus, particularly rhinovirus (14.0%), coronavirus E229 (12.4%), and influenza A(H3N2) virus (6.2%) while in Saudi Arabia. None of the participants were positive for the Middle East respiratory syndrome coronavirus. In addition, 50.0% and 62.0% of pre-Hajj and post-Hajj specimens, respectively, were positive for *Streptococcus pneumoniae* (p = 0.053). One third (36.3%) of the participants had acquired *S. pneumoniae* during their stay. Our results confirm high acquisition rates of rhinovirus and *S. pneumoniae* in pilgrims and highlight the acquisition of coronavirus E229.

## Abstract 2

The most common pathogens detected were coronaviruses, rhinoviruses, influenza viruses, and *Streptococcus pneumoniae*.

More than 2 million Muslims gather annually in Saudi Arabia for a pilgrimage to the holy places of Islam known as the Hajj. The Hajj presents major public health and infection control challenges. Inevitable overcrowding within a confined area with persons from >180 countries in close contact with others, particularly during the circumambulation of the Kaaba (Tawaf) inside the Grand Mosque in Mecca, leads to a high risk pilgrims to acquire and spread infectious diseases during their time in Saudi Arabia (*1*), particularly respiratory diseases (*2*). Respiratory diseases are a major cause of consultation in primary health care facilities in Mina, Saudi Arabia, during the Hajj (*3*). Pneumonia is a leading cause of hospitalization in intensive care units (*4*).

Numerous studies have shown a high prevalence of respiratory symptoms among pilgrims (*5*–*7*). Respiratory viruses, especially influenza virus, are the most common cause of acute respiratory infections among pilgrims (*8*–*11*). We recently reported the acquisition of rhinovirus (*5*) and *Streptococcus pneumoniae* infections (*12*) by French pilgrims during the 2012 Hajj season and highlighted the potential for spread of these infections to home countries of pilgrims upon their return. However, none of the French pilgrims were positive for Middle East respiratory syndrome coronavirus (MERS-CoV) in 2012 (*13*) and 2013 (*14*).

In this study, we collected paired nasal and throat swab specimens from adult pilgrims departing from Marseille, France to Mecca, Saudi Arabia, for the 2013 Hajj season. The primary objective was to determine the prevalence of the most common respiratory viruses and bacteria upon return of pilgrims from the Hajj. The secondary objective was to evaluate the potential yearly variation of the acquisition of these respiratory pathogens by comparing results from the 2012 and 2013 Hajj seasons.

## Methods

### Participants

Pilgrims who planned to participate in the 2013 Hajj were recruited on September 15, 2013, at a private specialized travel agency in Marseille, France, which organizes travel to Mecca. Potential participants were asked to participate in the study on a voluntary basis if they were ≥18 years of age and were able to provide consent.

### Study Design

In this prospective cohort study, participants were sampled and followed up before departing from France (on October 2, 2013) and immediately before leaving Saudi Arabia (on October 24, 2013). Upon inclusion in the study, participants were interviewed by Arabic-speaking investigators who used a standardized pre-travel questionnaire that collected information on the demographic characteristics and medical history of each participant. A post-travel questionnaire that collected clinical data and information on vaccination status and compliance with preventive measures was completed during a face-to-face interview 2 days before the pilgrims returned to France by a single investigator who joined the pilgrims after the Hajj. Health problems that occurred during the pilgrims’ stay were also recorded by a physician who traveled with them during the entire stay in Saudi Arabia, including during the rituals.

Subjective fever was defined as a feverish feeling according to the pilgrims’ report. Influenza-like illness (ILI) was defined as the presence of cough, sore throat, and subjective fever (*15*). The study protocol was approved by the Aix Marseille Université institutional review board (July 23, 2013; reference no. 2013-A00961–44) and by the Saudi Ministry of Health Ethical Review Committee. The study was performed in accordance with the good clinical practices recommended by the Declaration of Helsinki and its amendments. All participants gave written informed consent.

### Respiratory Specimens

Paired nasal and throat swab specimens were collected from each participant by using rigid cotton-tipped swab applicators (Medical Wire and Equipment, Corsham, UK) 10 days (September 22, 2013) before participants departed from France (pre-Hajj specimens) and only 1 day (October 23, 2013) before they left Saudi Arabia (post-Hajj specimens). Nasal and throat swab specimens collected from participants were placed in viral transport media (Virocult and Transwab, respectively; Sigma, St. Louis, MO, USA) at the time of collection and kept at 20°C before being transported to a laboratory in Marseille for storage at −80°C within 48 h of collection.

### Detection of Respiratory Viruses

Nasal swab samples were independently tested as described (*5*) for influenza virus A/H3N2 (*16*), influenza B virus (*16*), influenza C virus (*17*), and A(H1N1)pdm09 virus (*18*); human adenovirus (*19*); human bocavirus (*20*), human cytomegalovirus (*21*); human coronaviruses (HCoVs); human enterovirus (*22*); human metapneumovirus (*23*); human parainfluenza viruses (HPIVs); human parechovirus (*24*); human respiratory syncytial virus (*25*); and human rhinovirus (HRV) (*26*) by using real-time reverse transcription PCRs. HCoVs and human HPIVs were detected by using an HCoV/HPIV R-Gene Kit (Argene/bioMérieux, Marcy l’Etoile, France) (*27*). HCoV-positive samples were then genotyped by using the FTD Respiratory Pathogens 21 Kit (Fast Track Diagnostics, Luxembourg, Luxembourg).

### Detection of Respiratory Bacteria

Throat swab samples were independently tested as described (*12*) by using quantitative real-time PCRs for *Streptococcus pneumoniae*, *Neisseria meningitidis*, *Bordetella pertussis*, and *Mycoplasma pneumoniae*. Sequences of all primers and probes have been reported (*28*). In the present study, reactions were performed by using a 7900HT Fast Real-Time PCR System (Applied Biosystems, Foster City, CA, USA).

### Statistical Analysis

The Pearson χ^2^ and Fisher exact tests, as appropriate, were used to analyze categorical variables. Statistical analyses were performed by using SPSS software package version 17 (SPSS Inc., Chicago, IL, USA). p values ≤0.05 were considered significant.

## Results

### Characteristics of Study Participants

A total of 129 persons were invited to participate in the study. All persons agreed to participate in the study and responded to the pre-travel questionnaire. The participants were 77 women (59.7%) and 52 men (40.3%) who had a mean (SD) age of 61.7 (9.8) years (age range 34–85 years) (Table 1). Although most (94.6%) participants were born in northern Africa, most (94.5%) had lived for years in Marseille or the surrounding cities. More than half of the participants (52.7%) reported having ≥1 chronic disease, as described (*14*).

**Table 1:** Demographic and baseline characteristics of pilgrims during the 2012 and 2013 Hajj*

Characteristic	2012 study cohort, n = 169	2013 study cohort, n = 129	p value
Mean age, y (SD, range)	59.3 (12.4, 21–83)	61.7 (9.8, 34–85)	0.079
Age groups, y, no. (%)			
20–40	13 (7.8)	5 (3.9)	NA
41–60	67 (40.1)	50 (38.8)	NA
61–80	85 (50.9)	71 (55.0)	NA
>80	2 (1.2)	3 (2.3)	NA
Sex, no. (%)			0.669
M	64 (38.3)	52 (40.3)	NA
F	103 (61.7)	77 (59.7)	NA
Birthplace, no. (%)			
Algeria	116 (69.5)	90 (69.8)	NA
Tunisia	17 (10.2)	17 (13.2)	NA
Morocco	15 (9.0)	15 (11.6)	NA
Metropolitan France	13 (7.8)	5 (3.9)	NA
Egypt	6 (3.6)	2 (1.6)	NA
Location of residence in France, no. (%)			
Marseille	110 (65.9)	91 (70.5)	NA
Southern France (outside Marseille)	48 (28.7)	31 (24.0)	NA
Other	7 (5.4)	7 (5.4)	NA
Duration of stay in France, y , no. (%)†			
5–10	8 (5.4)	13 (11.1)	NA
11–20	13 (8.8)	11 (9.4)	NA
>20	127 (85.8)	93 (79.5)	NA

*NA, not applicable. †Immigrants only.

### Clinical Features

All post-travel questionnaires were completed. During the 3-week stay in Saudi Arabia (October 3–24, 2013), most (90.7%) pilgrims had ≥1 respiratory symptom, including cough (86.8%), sore throat (82.9%), rhinorrhea (72.1%), myalgia (50.4%), fever (49.6%), and dyspnea (21.7%), and 47.3% met the criteria for self-reported ILI (41.3% in 2012 vs. 47.3% in 2013; p = 0.325). Onset of respiratory symptoms peaked in the second week (week 41) after the arrival of the pilgrims in Mecca and decreased thereafter. However, 90 (69.8%) pilgrims still had respiratory symptoms before leaving Saudi Arabia at the time of sampling (week 43). Only 1 pilgrim (0.8%) was hospitalized during the stay in Saudi Arabia (for undocumented pneumonia). No deaths occurred.

Regarding preventive measures, 51.2% of participants reported receiving pneumococcal vaccination (Pneumo 23) in the past 5 years, which was significantly higher than the rate in 2012 (35.9% in 2012 vs. 51.2% in 2013; p = 0.013). None had received the 2013 influenza vaccine before departing for the Hajj, but 44.2% reported having received the seasonal influenza vaccine in 2012 (31.8% among participants <65 years of age vs. 65.8% among participants >65 years of age; p = 0.001). During the stay in Saudi Arabia, 53.5% of pilgrims reported either frequent use (9.3%) or occasional use (44.2%) of facemasks; 93.0% used disposable handkerchiefs; 49.6% reported frequent handwashing; and 67.4% used hand sanitizer. ILI symptoms were less frequently reported by persons who reported receiving the influenza vaccine in 2012 compared with reports by unvaccinated persons (34.1% vs. 61.5%, respectively; p = 0.009) (odds ratio 0.32, 95% CI 0.14–0.76). In contrast, none of the other preventive measures was found to be effective in preventing ILI symptoms during the stay in Saudi Arabia.

### Detection of Respiratory Viruses

**Figure 1:**
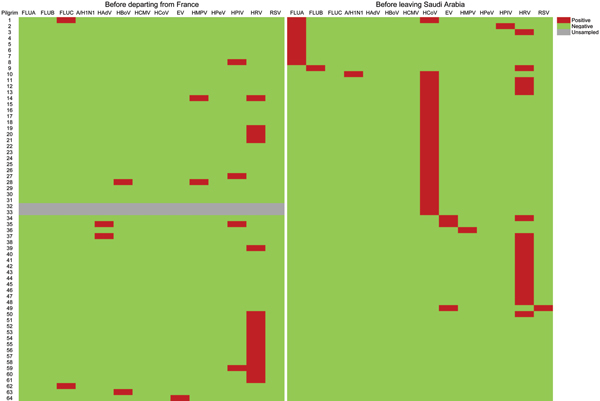
Patterns of respiratory viruses detected among 64 pilgrims who were positive for viruses during the study period before departing from France and before leaving Saudi Arabia, 2013 Hajj. FLUA, influenza A(H3N2) virus; FLUB, influenza B virus; FLUC, influenza C virus; A/H1N1, A(H1N1)pdm09 virus; HAdV, human adenovirus; HBoV, human bocavirus; HCMV, human cytomegalovirus; HCoV, human coronaviruses; EV, human enterovirus; HMPV, human metapneumovirus; HPeV, human parechovirus; HPIV, human parainfluenza virus; HRV, human rhinovirus; RSV, human respiratory syncytial virus;

Pre-Hajj and post-Hajj nasal swab specimens were obtained from 121 (93.8%) and 129 (100%) participants, respectively. A total of 26 (21.5%) of 121 pre-Hajj specimens tested were positive for ≥1 virus compared with 50 (38.8%) of 129 post-Hajj specimens tested (p = 0.003) (Table 2). Moreover, 36 (29.8%) participants had acquired ≥1 virus during the stay in Saudi Arabia (Figure 1). The prevalence of human coronavirus E229 (HCoV-E229) was significantly higher in post-Hajj specimens than in pre-Hajj specimens (12.4% vs. 0%; p<0.001). A high prevalence of HRV was observed in pre-Hajj and post-Hajj specimens (14.0% and 14.7%, respectively; p = 0.88). Of 19 participants whose post-Hajj specimens were positive for HRV, 17 (89.5%) had acquired the infection during their stay in Saudi Arabia (Figure 1).

**Table 2:** Prevalence of respiratory viruses and bacteria among participants before departing from France and before leaving Saudi Arabia, 2012 and 2013 Hajj*

Respiratory pathogen	2012 study, n = 169			2013 study, n = 129		
	Before departing from France, no. (%)	Before leaving Saudi Arabia, no. (%)	p value	Before departing from France, no. (%)	Before leaving Saudi Arabia, no. (%)	p value
Virus						
Influenza virus A (H3N2)	0	0	NA	0	8 (6.2)	0.007†
Influenza virus B	0	2 (1.3)	0.23	0	1 (0.8)	1
Influenza virus C	1 (0.6)	0	1	2 (1.7)	0	0.23
A(H1N1)pdm09	0	0	NA	0	1 (0.8)	1
Human adenovirus	1 (0.6)	3 (1.9)	0.36	2 (1.7)	0	0.23
Human bocavirus	ND	ND	NA	2 (1.7)	0	0.23
Human coronavirus E229	ND	ND	NA	0	16 (12.4)	<10^–3^†
Human coronavirus HKU1	ND	ND	NA	0	5 (3.9)	0.06
Human coronavirus NL63	ND	ND	NA	0	1 (0.8)	1
Human coronavirus OC43	ND	ND	NA	0	5 (3.9)	0.06
Human cytomegalovirus	ND	ND	NA	0	0	NA
Human enterovirus	1 (0.6)	1 (0.6)	1	1 (0.8)	3 (2.3)	0.62
Human metapneumovirus	0	0	NA	2 (1.7)	1 (0.8)	0.61
Human parainfluenza viruses	ND	ND	NA	4 (3.3)	1 (0.8)	0.20
Human parechovirus	ND	ND	NA	0	0	NA
Human respiratory syncytial virus	0	0	NA	0	1 (0.8)	1
Human rhinovirus	5 (3.0)	13 (8.4)	0.036†	17 (14.0)	19 (14.7)	0.88
At least 1 virus	8 (4.3)	17 (11.0)	0.040†	26 (21.5)	50 (38.8)	0.003†
Bacteria‡						
* Bordetella pertussis*	0	0	NA	0	0	NA
* Mycoplasma pneumoniae*	0	0	NA	0	0	NA
* Neisseria meningitidis*	0	0	NA	0	0	NA
* Streptococcus pneumoniae*	12 (7.3)	30 (19.5)	0.001†	63 (50.0)	80 (62.0)	0.053

*In 2012, 146 pre-Hajj samples (88.5%) were collected in the month before departing from France and were stored at ambient temperature (stabilized by air conditioning at 20°C) for a mean time of 13 d (range 5–37 d) before being transported to a laboratory in Marseille for storage at −80°C. In addition, 19 pre-Hajj samples (11.5%) were collected on the day of departure from France (at the airport) and were stored at ambient temperature for 30 d after collection before being transported to a laboratory in Marseille for storage at −80°C. In 2013, all samples collected during the study were kept at ambient temperature before being transported to a laboratory in Marseille for storage at −80°C within 48 h of collection. NA, not applicable; ND, not determined. †Statistically significant difference. ‡In the 2012 study, nasal swab specimens were collected from participants instead of throat swab specimens, which were used in the present study conducted in 2013.

The prevalence of influenza A and B viruses was significantly higher in post-Hajj specimens than in pre-Hajj specimens (7.8% vs. 0%; p = 0.002); further details are described elsewhere (*14*). Coronaviruses HKU1, NL63, and OC43; human enterovirus; human metapneumovirus; HPIV; and human respiratory syncytial virus were also acquired during the stay in Saudi Arabia by a low proportion of participants (Table 2). Of 50 participants whose post-Hajj specimens were positive for ≥1 respiratory virus, 43 (86.0%) reported ≥1 respiratory symptom during their stay in Saudi Arabia, of whom 37 (86.0%) still had respiratory symptoms at the time of sampling. Also, of 79 participants whose post-Hajj specimens were negative for respiratory viruses, 74 (93.7%) reported ≥1 respiratory symptom during their stay Saudi Arabia, of whom 53 (71.6%) still had respiratory symptoms at the time of sampling. None of the preventive measures was found to be effective in preventing respiratory viruses in post-Hajj specimens.

### Detection of Respiratory Bacteria

Pre-Hajj and post-Hajj throat swab specimens were obtained from 126 (97.7%) and 129 (100%) participants, respectively. None of the participants were positive for *N. meningitidis*, *B. pertussis*, or *M. pneumoniae* at any point in the study period (Table 2).

**Figure 2:**
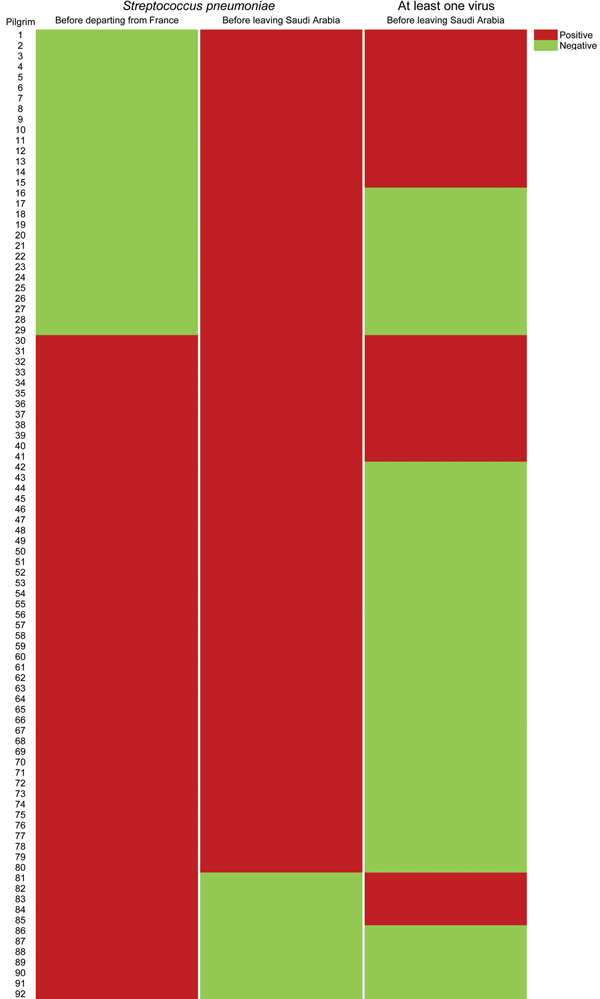
Detection of *Streptococcus pneumoniae* among 92 pilgrims who were positive for viruses during the study period before departing from France and before leaving Saudi Arabia, 2013 Hajj.

A total of 63 (50.0%) of 126 pre-Hajj specimens tested and 80 (62.0%) of 129 post-Hajj specimens tested were positive for *S. pneumoniae* (p = 0.053) (Table 2; Figure 2). Of 80 participants whose post-Hajj specimens were positive for *S. pneumoniae*, 29 (36.3%) had acquired the infection during their stay in Saudi Arabia (Figure 2). In addition, of 63 participants whose pre-Hajj specimens were positive for *S. pneumoniae*, 12 (19.0%) subsequently had post-Hajj specimens that were negative for *S. pneumoniae* (Figure 2), of whom 10 (83.3%) reported having received antimicrobial drugs during their stay in Saudi Arabia: 7 received amoxicillin, 2 received amoxicillin and ciprofloxacin, and 1 received azithromycin. Of 80 participants whose post-Hajj specimens were positive for *S. pneumoniae*, 73 (91.2%) reported ≥1 respiratory symptom during their stay in Saudi Arabia, of whom 56 (76.7%) still had respiratory symptoms at the time of sampling.

Among 66 participants who reported having received a pneumococcal vaccination in the 5 years before traveling to Saudi Arabia, 37 (56.1%) had post-Hajj specimens that were positive for *S. pneumoniae*. The prevalence of *S. pneumoniae* in post-Hajj specimens was significantly lower in persons who reported using hand sanitizer during their stay in Saudi Arabia than in remaining participants (55.2% vs. 76.2%; p = 0.021) (odds ratio 0.39, 95% CI 0.17–0.88) and slightly lower in persons who reported more frequent handwashing than usual during their stay in Saudi Arabia than in persons who reported usual handwashing (54.7% vs. 69.2%; p = 0.08).

Of 80 participants whose post-Hajj specimens were positive for *S. pneumoniae*, 27 (33.8%) were co-infected with ≥1 virus (Figure 2). Of 49 participants whose post-Hajj specimens were negative for *S. pneumoniae*, 23 (46.9%) were infected with ≥1 virus (33.8% vs. 46.9%; p = 0.14) (Figure 2).

## Discussion

For the second consecutive year, we conducted a prospective longitudinal study of respiratory viruses and bacteria in respiratory specimens collected from a single cohort of pilgrims before departing from Marseille, France, to Mecca, Saudi Arabia, for the Hajj and immediately before leaving Saudi Arabia. By collecting samples from pilgrims before their departure from Saudi Arabia, we were able to rule out acquisition of infections acquired as a result of travel through the international airports of Jeddah, Saudi Arabia, and Istanbul, Turkey, as part of the return trip to Marseille. Close monitoring for respiratory symptoms and compliance with preventive measures was also performed by the investigators accompanying the group.

In this study, we confirmed that performing the Hajj pilgrimage is associated with an increased occurrence of respiratory symptoms in most pilgrims; 8 of 10 pilgrims showed nasal or throat acquisition of respiratory pathogens. This acquisition may have resulted from human-to-human transmission through close contact within the group of French pilgrims because many of them were already infected with HRV or *S. pneumoniae* before departing from France. Alternatively, the French pilgrims may have acquired these respiratory pathogens from other pilgrims, given the extremely high crowding density to which persons from many parts of the world are exposed when performing Hajj rituals. Finally, contamination originating from an environmental source might have played a role. Sequencing of these pathogens would be required to determine how often new infections were acquired during the stay in Saudi Arabia. However, detection of nasal carriage of coronaviruses other than MERS-CoV and influenza A and B viruses in only the post-Hajj specimens supports the hypotheses that infection occurred during the Hajj.

We confirmed the predominance of HRV and *S. pneumoniae* among pathogens acquired during the pilgrims’ stay (*5*,*12*). We also highlighted acquisition of coronaviruses other than MERS-CoV, most notably HCoV-E229, by pilgrims during the 2013 Hajj pilgrimage. In 2012 and 2013, results of screening for MERS-CoV infection in different cohorts of pilgrims, including the present cohort, were negative (*13*,*14*,*29*). Finally, we found that compared with acquisition of HRV and HCoV-E229, influenza viruses were acquired at a lower frequency among pilgrims.

The present study is a continuation of our previous study in 2012 (*5*). We extended the investigation to additional viruses, including human bocavirus, human cytomegalovirus, coronaviruses, human parechoviruses, and HPIV, and showed a high frequency of HCoV-E229 infection in pilgrims returning from the Hajj. The prevalence of HRV was lower in 2012 than in 2013, both before departing from France (3.0% in 2012 vs. 14.0% in 2013; p = 0.001) and before leaving Saudi Arabia (8.4% in 2012 vs. 14.7% in 2013; p = 0.092). However, samples that were obtained from pilgrims before departing from France during the 2012 study were stored at room temperature (20°C) for ≤30 days before being processed. This protocol may have resulted in degradation of genetic material, which probably contributed to underestimation of frequencies of infection in 2012. In 2013, all samples collected during the study period were stored at −80°C within 48 h of collection.

The prevalence of *S. pneumoniae* was also significantly lower in 2012 than in 2013 before pilgrims departed from France (7.3% vs. 50.0%; p<0.001) and before they left Saudi Arabia (19.5% vs. 62.0%; p<0.001). However, in the 2012 study, nasal swab specimens were collected from participants instead of throat swab specimens, which were used in the 2013 study. In addition, the period of the storage of samples before freezing differed between the 2012 and the 2013 studies, as mentioned earlier in this report.

Our results confirm that various respiratory viruses might be acquired by pilgrims during their stay in Saudi Arabia and introduced into home countries of pilgrims on their return, thus contributing to potential international spread of these viruses. However, detection of other human coronaviruses does not enable any conclusions regarding MERS-CoV, for which the available data to date, although limited, indicate different epidemiologic characteristics. We could not demonstrate whether pathogens detected in respiratory specimens were responsible for observed symptoms because nasal carriage was observed in asymptomatic pilgrims in certain instances, and symptoms might have resulted from infection by pathogens that were not investigated in our study. In future studies, checking pilgrims at more frequent intervals might provide useful information. Nevertheless, we believe that Hajj cough likely results from infection of the respiratory tract by various respiratory viruses, including HRV and HCoV-E229, which are known to cause mild or serious lower respiratory tract infections (*30*,*31*). However, our results cannot be extrapolated to all pilgrims. A large-scale study based on a similar design and conducted in a large number of pilgrims from many countries would be useful.

We found that pilgrims who had received influenza vaccine in 2012 were less likely to report ILI symptoms during their stay in Saudi Arabia in 2013. Thus, availability of seasonal influenza vaccine for all persons attending the Hajj is crucial. Vaccination with a conjugate pneumococcal vaccine should be considered for persons with medical risk factors for invasive pneumococcal disease. In addition, use of hand sanitizer during the stay in Saudi Arabia was reported by more than two thirds of pilgrims in our survey and was associated with a lower prevalence of *S. pneumoniae* carriage. Interventional studies are urgently needed that evaluate efficacy of influenza and pneumococcal vaccines and use of hand sanitizer and closely monitor respiratory symptoms and carriage of respiratory pathogens in large cohorts of pilgrims. It is expected that results of such studies will lead to implementation of evidence-based recommendations about preventive measures during the Hajj.
